# Alcoholic Dressing of Wounds, as a Phophylactic against Erysipelas

**Published:** 1879-07

**Authors:** Alfred Emanuel Regensberger


### ALCOHOLIC DRESSING OF WOUNDS, AS A PHOPHYLACTIC AGAINST ERYSIPELAS.

By ALFRED EMANUEL REGENSBERGER, M.D.

Erichsen, the great English clinical master and illustrious surgeon, in his work on the Art and Science of Surgery, Chapter XXXII, page 443, expresses himself as follows : "Erysipelas is an affection that so frequently and suddenly complicates most other surgical injuries, that its study is of the utmost importance to the practical surgeon." This is not his opinion alone ; it simply coincides with that of every surgeon. It is, consequently, obvious that any means or method of treatment which prophylactically or otherwise, lessens the danger or insures immunity from it, is well worthy of a few months attention ; and still more so, when we remember that the advocate of the method presently to be described, also claimed that it prevents to a great extent the occurrence of pyemia, phlebitis, and other dreaded constitutional affections which succeed surgical injuries and operations. As it would take up too much space to enter upon alcholic dressings of wounds in general, I shall limit myself so that I shall only touch upon them as far as they have reference to erysipelas.

While sojourning in Paris, where I was a frequent visitor of Professor Gosselin's surgical clinic at La Charite, I was struck by the fact of the very rare occurrence, if not entire absence, of erysipelatous manifestations after surgical injuries and operations. I was not long allowed to remain in the dark concerning this very important matter, for the professor repeatedly stated that while he had treated wounds with alcoholic dressings, his wards had been free from the diseases in question. Only one case occurred, to my knowledge, during my six months' residence in the capital of the world ; the subject being a young man who was admitted to his wards, suffering from a wound of the scalp, and subsequently developed erysipelas. It must, however, be said that this case was hardly a fair criterion, and should absolutely carry no weight with it, as the alcoholic dressing was applied by outside physicians, and it was not known whether it had been properly applied, or whether the patient had followed the physician's advice in relation to it, even if properly used. It is simply mentioned for completeness's sake. The good results of this treatment were not restricted to this hospital des Cliniques—reputed one of the most insalubrious of Paris. After the great Nelaton had introduced this method of treatment at the last named institution, there was a marked abatement of this and other diseases which are wont to follow surgical procedures and wounds, and which had been so prevalent. From that time onward its real value became pretty thoroughly recognized in France, its opponents not being able to entirely deny its usefulness. Professor Dolbeau, a most talented surgeon and erudite physician, could only state that he had used it for some time at the Hospital St. Louis, and did not believe in its vaunted superiority over all other methods of dressing wounds; yet that it was equally true that it was preferable to a great many others. If such good results were obtained in less favored climes than ours, we certainly have something to expect from it. We can derive some further encouragement, as a few members of the profession in this city have used it, and are using it (as I am informed), which certainly tends to indicate that they have derived some advantage from its employment, or else they would have abandoned it.

It is true that it is nothing new, but that should not detract anything from its merits. It requires no great learning or profound erudition to know that alcohol was used by the ancients for dressing wounds. In the numerons formulæ used for this purpose, alcoh ol very often formed the base. It entered into those of Ambrose Pare, Hippocrates, and others. However, it rarely was used alone, or in the form of the *eau de vie camphree* as is the case at present in France.

As regards its action in preventing erysipelas, I do not wish to spin any fine theories, or give those of others. It is well-know that alcohol has long been used for preserving anatomical preparations. It may perhaps act here in the same manner as it does there, viz., it prevents putrefaction by destroying the vitality and preventing the ingress of those germs upon which putrefactive and other changes depend. Be that as it may, it possesses three advantages over many other dressings which may be briefly formulated—1st, simplicity; 2d, cleanliness; 3d, absence of all odor. Unquestionably, something that cannot be said of many of the complicated dressings in use at the present day. To convince one's-self of the correctness of the above assertion, having used or having seen it properly employed for a very short space of time, is all that is requisite. In applying this dressing all that is necessary is some alcohol and some charpie—no spray-producers or other elaborate or costly paraphernalia. The piece of charpie is moistened with alcohol, and the wound, after being otherwise properly arranged on general surgical principles, is covered with it. The dressing is changed two or three times in 24 hours, care being taken to keep the charpie continually saturated with alcohol. In France a great many use *eau de vie camphoree* instead of alcohol alone. Whether the camphor adds anything to the efficacy of the alcohol I am not prepared to say, but I am rather inclined to think that it does not. Contrary to what one might suppose, the pain occasioned by the application of alcohol to a wound is not severe. The first two or three applications cause a slight smarting sensation, after which it is *nil.* Possessing all these advantages, and very few drawbacks, we trust that this method may receive a trial by the profession on this coast, and if it responds to the hopes entertained of it, that it may be used universally.—*Western Lancet.*

